# Groove or swing as distributed rhythmic consonance: introducing the groove matrix

**DOI:** 10.3389/fnhum.2014.00454

**Published:** 2014-06-23

**Authors:** Bjorn Merker

**Affiliations:** Independent scholarKristianstad, Sweden

**Keywords:** entrainment, isochrony, groove, rhythmic harmonic series, predictive timing, swing, synchronization

Groove or swing are terms employed in popular music genres to designate the efficacy of rhythmic musical structures in motivating us to move in time to their beat (pulse, tactus, for which see Arom, 1991, p. 179 ff). Precise on-the-beat synchronization of bodily movement to such structures is only possible through predictive timing, for which the regular periodicity—isochrony—of the pulse provides essential perceptual support (Fraisse, 1982; Merker et al., 2009). It makes the next beat in the sequence perfectly predictable, enabling bodily entrainment to the isochronous signal, most readily so at tempos centered on two cycles per second (120 beats per minute), which is also the human locomotor tempo (Fraisse, 1982; MacDougall and Moore, 2005). Such entrainment features fluid interplay between two modes of timing control whose neural implementation appears to depend on cerebellum plus sensorimotor cortex and the fronto-parietal attention network respectively (for which see Merker et al., 2009, pp. 11–12; see also Lewis and Miall, 2003).

Not all musical structures based upon an isochronous pulse are equally effective in motivating entrainment to their beat, however. This allows groove to be defined as a perceptual dimension by means such as rating scales (Madison, 2006; Janata et al., 2012), and has occasioned speculation regarding its structural basis, typically in terms of systematic “deviations from isochrony” in the relative timing of structural elements of rhythm (Keil, 1966; Bengtsson, 1975; p. 342: “pulling against the pulse”; Keil and Feld, 1994, p. 155: (music must be) “out of time to groove”). This idea has been explored experimentally, most often with regard to the “swing” phenomenon in jazz (Keil, 1987, 1995; Prögler, 1995; Collier and Collier, 1996; Busse, 2002; Friberg and Sundström, 2002; Iyer, 2002; McGuiness, 2005; Honing and De Haas, 2008; Polak, 2010), not always faring well on empirical scrutiny (Butterfield, 2011; Wesolowski, 2012; Davies et al., 2013).

The claim that deviations from isochrony constitute the phenomenon of groove or swing is so counter-intuitive as to be tantamount to a contradiction in terms. It asks us to believe that our motivation to engage in predictive synchrony is driven by structural musical content that deviates from, and thus potentially dilutes, obscures, or detracts from, the causal key to that synchrony, which is the isochrony that serves as its predictive basis and target. Intuition is supported by empirical findings that contradict an account of groove in terms of deviations from isochrony (Davies et al., 2013).

An alternative to construing groove in terms of deviations from isochrony is provided by a principle that specifies the conditions under which complex rhythmic timing relations come to form a *global* constellation that reinforces rather than detracts from the isochronous pulse. On intuitive grounds alone it would seems that groove or swing should benefit from having the interval between the beats of the tactus occupied not by time markers that deviate from the prediction-framework of pulse isochrony, but by events whose placement supports that framework by being consonant with it.

There is in fact a comprehensive formal source of canonical positions that fill this requirement. Just as in the domain of pitch the half-dozen periodicities that occupy the bottom end of the harmonic series remain consonant when collectively sounding together, so their exact discretized analogs in the domain of rhythm (see Figure 1) do not interfere with one another rhythmically, but yield a rhythmically coherent—rhythmically consonant—global pattern when playing in parallel.

**Figure 1:**
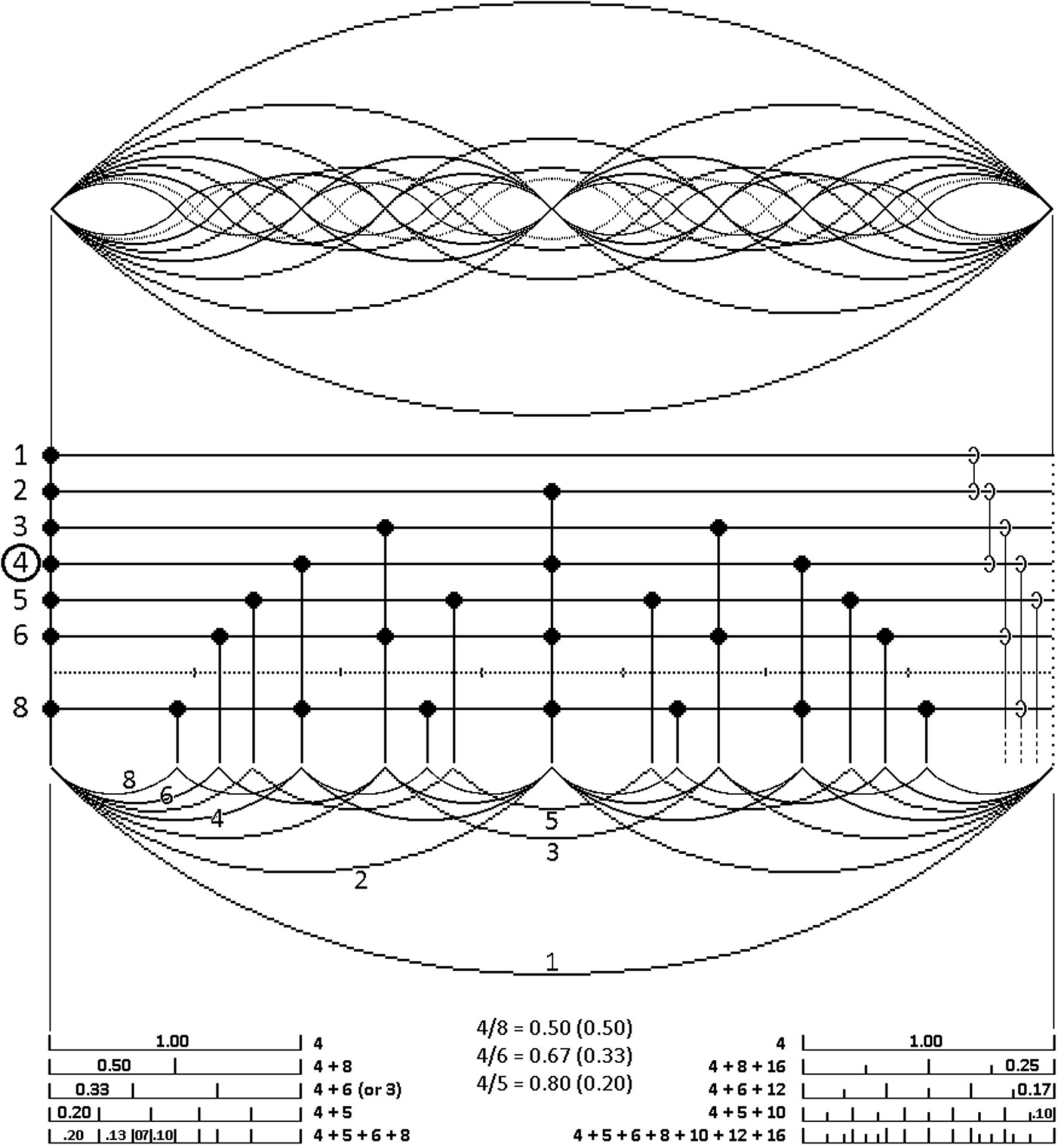
**The first eight harmonic vibrational modes of a string, displayed in their entirety in the top part of the figure (where the seventh harmonic is rendered by a dotted line)**. Split along its zero-crossings, half of this set of nested sine waves appears beneath the intervening grid, showing how sine wave zero-crossings yield a corresponding set of isochronous point processes. Time cycles from left to right. Omitting the seventh harmonic (see text) makes the contents of the grid a “five-limit” system of harmony for rhythm. For illustrative purposes (yielding a four-beat rhythmic cycle) the fourth harmonic is chosen as the level of the pulse, assumed to lie around 2 Hz. It is marked by a circle on the discretization grid, whose right-hand margin depicts octave relations and their (dashed) continuation to higher octaves. The subdivision of the pulse period by the other harmonics is summarized in the diagrams at the bottom of the figure.

A musical pulse can be conceived of as an isochronous point process derived by discretization from the zero-crossings of a pure tone of suitably low periodicity, say around 2 Hz (see above). As an illustrative exercise, tailored for convenience to a four-beat rhythmic cycle as in Figure 1, construe such a 2 Hz periodicity as the fourth harmonic (counting the fundamental as the first harmonic) of a fundamental periodicity of 0.5 Hz, repeating indefinitely. All its harmonics (i.e., 0.5, 1.0, 1.5, **2.0**, 2.5, 3.0, 3.5, 4.0 Hz, etc.) are isochronous point processes in their own right, and form an infinite harmonic series of isochronies strictly analogous to the harmonic series of pitches (for the parallelism between pitch and rhythm, see Monahan, 1993).

Just like pitches, these harmonic isochronies can be added to one another to play in parallel. When started at the same point in time, the timing differences of their unique periodicities create composite joint patterns repeating (in this case) every four beats, specific to each additional harmonic brought into play. Thus the 0.5 Hz “first harmonic” fundamental plus its first two even harmonics (the second of which is the pulse in our example) establish a repeating four beat and bi-partite rhythmic pattern through summation of point events that coincide in time across the harmonics (see the unequal number of vertically aligned event markers in Figure 1).

This four beat pattern is readily subdivided by point events at ever finer binary subdivisions of the inter-beat interval of the pulse (pulse “octaves”). These are all trivially compatible (“consonant”) with the pulse isochrony at 2.0 Hz, and correspond to successive octaves in the pitch domain. Although in principle one might add such pulse octaves indefinitely, soon the resulting event densities couple individual elements perceptually into highly salient “rolls,” effectively ending their independence as elements of combinatorial rhythmic figures.

Crucially, when the first couple of odd isochrony harmonics—the third and the fifth at 1.5 and 2.5 Hz straddling the 2.0 Hz pulse in our example—are added to the set of simultaneously playing pulse octaves, they create qualitatively new rhythmic patterns without disrupting rhythmic coherence. The sixth harmonic, being the octave of the third, is also compatible with the others. However, the fact that odd harmonics insert their events *between* those of *any* even one, generates a curious effect on the addition of the seventh isochrony harmonic (3.5 Hz in our example). The placement of its second and final elements with respect to their neighbors from other isochrony harmonics brings the “event density effect” into play, resulting in decelerated and accelerated “rolls” at the beginning and end of the sequence, even at tempos considerably below 120 bpm.

This “premature event density effect” makes the seventh isochrony harmonic problematic with regard to its utility for rhythmic pattern generation. The situation is curously reminiscent of the fact that in the pitch domain, consonance is maintained on the addition of the first couple of odd harmonics to the even ones, with growing perceptual dissonance taking hold as further odd harmonics are added to the simultaneously sounding set of pitches.

Expressed in the “limit” terminology of traditional harmony, this set of the first five isochrony harmonics plus any of their higher octaves constitutes a “five-limit” system of harmony for rhythm. I call it the groove matrix. Each isochrony of this system is mutually compatible with the pulse, with every other member isochrony, and with their aggregate. None of their combinations disrupt rhythmic coherence. In the aggregate this yields a rich set of isochrony-compatible subdivisions of the pulse period, numerically rendered for the first and second octave above the pulse at the bottom of Figure 1.

The density of these subdivisions makes the construct “deviation from isochrony” lose much of its definition. A note assumed to deviate from the sparser set of binary and ternary (triplet) subdivision dominating conventional music notation may in fact fall on a canonical subdivision of the richer groove matrix. This may apply, for instance, to various placements of the critical note of the so called “swing ratio” in jazz (Friberg and Sundström, 2002). Also, a number of “shuffle” variants resist definition in conventional notation (Bornemark, 2009). Central among these is a variant timed to the period of the 10th isochrony harmonic, i.e., the octave of the fifth harmonic in Figure 1 (for details, see section 5.1 of Hallström, 2000).

The structure depicted in Figure 1 does not itself “groove” (see below). Rather, it provides a formally defined set of intervals, a temporal “vocabulary,” for a combinatorics of rhythmic patterns potentially consonant with and thus supportive of the predictive synchronizing framework of an isochronous pulse. It supplies, I claim, a formal scaffolding for rhythmic music quite generally, but more particularly for genres dedicated to the aesthetics of groove or swing. Its basic structure has long since been captured in the comprehensive formal South Indian system of rhythm didactics, Konokol or Konnakol (see McLaughlin and Vinayakram, 2007 for an introduction). With its binary, ternary, and quinary subdivisions along with halving and doubling of tempo, Konokol embodies a five-limit system of rhythmic harmony as defined here.

The long-range sequence complexity of the multi-cycle “rhythmic dissertations” of Konokol virtuosos is not to be equated with groove, however. Groove is a far more short range and broadly accessible rhythmic quality that motivates entrainment to a rhythm in the listening “moment” (loosely construed as the span of auditory “echoic” memory of a few seconds, for which see Darwin and Turvey, 1972). If groove is a matter of *global* rhythmic constellations surrounding the pulse that reinforce rather than detract from its isochrony, how is the groove matrix deployed to generate such structures?

First, the inducing rhythmic pattern has to tie up much of our tremendous capacity for auditory scene analysis (Bregman, 1990) in patterns whose singular common denominator is the isochronous pulse. That is, the inducing pattern has to be rhythmically rich by drawing on many harmonic levels of the groove matrix (Madison, 2014; see also Hurley et al., in press). Second, the achievement of that richness must adhere to an economy of means by psychologically suggesting the most levels with the least amount of actual marking of time positions. Crucial in this regard is the fact that isochrony levels above and below the pulse period are rhythmically *coupled*.

To take a trivial example: the pulse octave (eighth harmonic, or eighth notes) is suggested by any event occurring half-way between two beats of the pulse with some regularity (at least once within the span of echoic memory). If marked only after every fourth beat of a plain pulse, the beats of the pulse are grouped into an implicit four beat cycle without the need to mark the first harmonic periodicity itself (the fundamental). Thus two harmonic levels have been added to the plain pulse by a single marked event. The result is the basic rhythm of the tango.

The principle of economy often leads to marking only some of the available positions on any harmonic level, perhaps even on that of the pulse itself. To skip the second pulse beat of a four beat cycle while marking a single eighth note before the next marked pulse beat is one of the most common rhythmic devices world-wide. Again, two harmonic levels (fundamental and pulse octave) have been added through a single omission-plus-marking (i.e., a dotted quarter note plus eighth note). The fact that the groove matrix itself violates this economy principle by marking every event on every harmonic level helps explain why it itself does not “groove.”

Extended across all the harmonic levels of the pulse matrix these principles open up a cornucopia of combinatorial possibilities for realizing rhythmic unity around the pulse through diversity of contributory patterns. The principles make groove a matter of filling the spaces between pulse beats with rhythmically optimal subdivision content. That means combinations of groove matrix elements which—within a running temporal span of a few seconds—support the isochrony of the pulse by the combinatorial parsimony with which they fill the spaces between its beats with events suggestive of remaining levels of the groove matrix. The polyrhythms of traditional African percussion ensemble music provide copious examples of this principle in practice (Arom, 1991; Hallström, 2000).

The groove matrix unlocks, I submit, the secret of groove or swing, an opinion that should be susceptible to empirical scrutiny given careful attention to the formal principles sketched here in all brevity.

## Conflict of interest statement

The author declares that the research was conducted in the absence of any commercial or financial relationships that could be construed as a potential conflict of interest.
